# Meeting of the Fulton County Medical Society

**Published:** 1914-11

**Authors:** R. R. Daly


### FULTON COUNTY MEDICAL SOCIETY.

Regular Meeting November 5, 1914.

By R. R. Daly, M. D.

Dr. E. C. Davis gave a paper on "Is Cancer on the Increase—Means For Its Correction."

Dr. L. C. Fischer said he had heard Coker say that he had operated upon more cancer growths than tubercular, during the last twelve months. As to immune people, Bainbridge said that Russian Jews are not meat eaters and they are nearly free from cancer. Among men he would say that cancer was on the increase. One out of every six men after thirty-five years of age has cancer.

Dr. R. R. Kime said that it doesn't seem as if we had made much advance in the treatment of malignant conditions except that they must be removed early and thoroughly searched for whenever there is the least indication of their presence. Among women, curettage after 45 when there is recurrent menstruation will often show malignancy. Continual irritation of any part leads toward cancer. A great deal depends upon the general condition of the patient. As a nation, we live under large stresses and without proper care of our diet. Thus we are prone to trouble. To prevent it, we must try to be normal in our lives and then to remove all foci of chronic irritation wherever they may be.

Dr. R. M. Nelson read a paper, "A Suggestion of Phenol and Ichthyol in Otitis Externa."

Dr. R. R. Daly said that he had used the combination of Phenol and Ichthyol each 5% in Glycerine at Nelson's suggestion and had met with success. It had shortened cases of furunculosis of the canal at his clinic and had been of benefit in other infectious states.

Dr. Nelson said that in Panama in 1906-07, there had been an epidemic of Otitis externa which seemed to come from the bathing. The treatment had been of great value to him at that time.

Dr. F. W. McRae exhibited two cases in which the diagnosis was not clear until after the laboratory findings. One was a case of stone in the kidney that had been thought to be appendicitis. Ureteral catheterization showed pus from the right kidney which had apparently given no trouble and was not suspected. The other case showed small stone in pelvis of kidney. There was no obstruction or loss of function. The x-Ray showed it.

### REGULAR MEETING NOVEMBER 19, 1914.

Dr. Allen H. Bunce read a paper, "The Diagnostic Value of Examination of the Spinal Fluid." It appears in the Journal.

Dr. C. W. Gould said that in examining cells, it is well to note their character and age by proper staining. Lange's colloidal test consists of ten tubes of different strength of coloring, and the number of these that is necessary to use in staining the various cells determines the age of the cells and shows whether they are from the brain or cord. It is a beautiful test and can readily be obtained.

Dr. Hansell Crenshaw said that he had had Bunce's aid in studying syphilis of the cord and brain and that it has been a satisfaction to have these measurements and counts accurately made. He found them of great value five times in one week in one case. We do not make punctures often enough to secure our diagnoses.

Dr. L. M. Gaines said he had been interested in the spinal fluid for many years. A large number of cases demands its study in order to determine the diagnosis. For example, a child of five years was fretful and restless with little or no fever. The clinical symptoms were all negative except a peculiar cry that is heard with spinal troubles. A puncture was done. The pressure was 300 m. m. The cell count was high. Bunce found tubercle bacilli in the fluid and the diagnosis was complete.

It has both a positive and a negative value, in that it may show no organic disturbance is present and we can look for other things, as the functional trouble gives these symptoms. Its most important field is in syphilis. It is as important to examine the spinal fluid in this disease when it is suspected as it is to examine the urine when kidneys are likely to be involved. Every case of convulsion after 30 should have both puncture and a Wassermann. As to the puncture itself, sometimes it is easy and at others one wonders where the canal is concealed. The posterior ligament is sometimes cartilaginous and the intra spinous spaces are of different size. The body should always be bent well forward to get the interspace.

Dr. A. L. Fowler read a paper on "Spinal Anesthesia in Genito-Urinary Surgery." It appears elsewhere in the Journal.

Dr. W. B. Emery said that he had seen several of Fowler's cases. The method was either very good or of no use at all. That is, the anesthesia was so complete that the patient did not know he was being operated upon though he was talking at the time, or another anaesthesia had to be given to prevent the pain. It is of undoubted value in the hands of a specialist in the administration in diabetic cases where other anaesthetics are contraindicated. It should never be used when local anaesthesia can be successfully applied and always it should be in the hands of a man of experience.

Dr. E. P. Merritt said he was much interested in Fowler's success. It gave great promise of help in G. U. work.

Dr. Montague Boyd said that he was glad to see G. U. men branching out and taking cognizance of everything that could be of possible value to the specialty.

Dr. E. D. Highsmith said that he had spent much time in Vienna at Wertheimer's clinic and he had there seen cancer of the uterus removed under spinal anaesthesia as well as several other long operations. The anaesthesia departs in about fifty minutes and other things have to be given when the work lasts longer than that time. The Trendelenberg position had to be assumed very shortly after the injection which was given in the sitting posture. He did not see the after-results of any of the cases.

Dr. M. C. Pruitt said he had seen thirty cases in that same clinic. It took a minute or more for the sensation to disappear. The patients often vomited upon the table. Besides this they were likely to be shocked by seeing the operation and their faces were usually covered.

Fowler said in closing that it took about ten minutes for anaesthesia to be complete. It began in the toes and crept upward gradually. There has been no post-operative pain. It has not been necessary to use the Trendelenberg position to protect the brain in his cases. The chief value of the method is in lame kidneys and in diabetes where operation is needed.

